# Levothyroxine treatment and incident dementia in adults with atrial fibrillation

**DOI:** 10.1007/s40520-019-01217-3

**Published:** 2019-05-22

**Authors:** Per Wändell, Axel C. Carlsson, Xinjun Li, Jan Sundquist, Kristina Sundquist

**Affiliations:** 1 Division of Family Medicine and Primary Care, Department of Neurobiology, Care Science and Society, Karolinska Institutet, Alfred Nobels Allé 23, 141 83 Huddinge, Sweden; 2 Center for Primary Health Care Research, Lund University, Malmö, Sweden; 3 Department of Family Medicine and Community Health, Icahn School of Medicine, Mount Sinai, NY USA; 4 Department of Population Health Science and Policy, Icahn School of Medicine, Mount Sinai, NY USA; 5 Department of Functional Pathology, School of Medicine, Center for Community-based Healthcare Research and Education (CoHRE), Shimane University, Matsue, Japan

**Keywords:** Atrial fibrillation, Alzheimer’s disease, Dementia, Gender, Drug treatment, Levothyroxine

## Abstract

**Objective:**

Levothyroxine treatment is common among older adults as is atrial fibrillation (AF), yet less is known about its potential effects on the development of dementia.

**Methods:**

The study population included all adults with diagnosed AF (*n* = 156,104) aged ≥ 45 years in Sweden without an earlier recorded diagnosis of dementia. Individuals with a dispensed prescription of levothyroxine on two or more occasions between July 1 2005 and December 31 2006 in Sweden were considered exposed (*n* = 12,978; 8.3%), and were compared to all other patients with AF without this treatment. Cox regression with hazard ratios (HRs) and 95% confidence interval (95% CI), with outcome defined as dementia of all causes between January 1, 2007 and December 31, 2015, was used in the analysis. Adjustments were made for socio-demographic factors (age, immigration status, marital status, educational level, neighborhood socioeconomic status), co-morbidity (cardiovascular disease, obesity, diabetes, COPD, depression, anxiety and alcohol related diagnoses), and cardiovascular medications.

**Results:**

During follow-up, a total of 9054 patients with AF were diagnosed with dementia (5.8%). We found no significant association of levothyroxine treatment and incident dementia, fully adjusted HR 1.03 (95% CI 0.96–1.11), neither among men and women, nor in different age-groups or subgroups of dementia.

**Conclusion:**

We found no significant association of levothyroxine treatment and incident dementia among patients with AF, which contrasts some earlier findings.

**Electronic supplementary material:**

The online version of this article (10.1007/s40520-019-01217-3) contains supplementary material, which is available to authorized users.

## Introduction

There are conflicting results on the association between thyroid function and incident dementia. An increased risk of dementia in patients with hypothyroidism has been found in some studies. For example, one study of patients with Alzheimer’s dementia (AD) found hypothyroidism to be overrepresented among those patients with dementia [1], while another study did not find hypothyroidism to be associated with AD pathology but instead with cerebrovascular diseases and thus with vascular dementia (VaD) [2].

However, there are possible mechanisms supporting the association between hypothyroidism, thyroid hormones and dementia. Thyroid hormones certainly are involved in the development and maturation of the brain in early life, and the lack of thyroid hormones has deleterious effects on the brain in congenital hypothyroidism [3, 4]. Thyroid hormones could also exert an effect on the adult brain, e.g. showing a positive effect on memory in euthyroid men [5], and with thyroid hormones showing improvement in hippocampus function [6], and an augmentation of cholinergic activity [7].

Atrial fibrillation (AF) is increasingly common with age and has also been shown to be associated with a higher dementia risk [8–10], where the most important factor is the increased risk of cardio-embolic stroke [10]. In accordance with this, anticoagulant treatment has been shown to be associated with a lower risk of dementia in patients with AF in observational studies [11, 12]. To use a population of patients with AF, as they are generally prescribed many cardiovascular drugs, is also a way to test if levothyroxine may affect the development of dementia in patients with complex cardiovascular pharmacotherapies. For example, antihypertensive drugs have been shown to reduce the risk of dementia, such as thiazides in combination with a RAS-blocker (ACE-inhibitor or angiotensin-receptor blockers, ARBs) in patients with AF [13], and calcium channel blockers and ARBs in older people [14].

The relation between hypothyroidism, levothyroxine, and mortality has been elucidated in earlier research, where hypothyroidism and levothyroxine treatment was associated with a lower cardiovascular risk and mortality [15], even if the potential mechanism explaining the association between levothyroxine treatment and mortality is unknown.

Women are known to exhibit a higher prevalence of hypothyroidism and thus of levothyroxine treatment than men [16]. Besides, women with AF are also found to have a higher relative risk of stroke than men [17]. Hence, men and women should be analyzed separately.

The primary aim was to study the association between levothyroxine treatment and dementia among patients with AF in Sweden.

## Methods

We examined information on individuals from Swedish population-based registers with national coverage. These registers were linked, using each person’s unique identification number, replaced by a serial number to preserve confidentiality. The study population was defined from the Swedish Total Population Register at Statistics Sweden, which also delivered the individual socioeconomic variables. The Inpatient Register, the Swedish Prescribed Drug Register and the Swedish Cause-of-Death Register were obtained from The National Board of Health and Welfare. These registers contain individual-level data on age, gender, education, hospital admissions, mortality and cause-of-death for all residents registered in Sweden. Ethical approvals were obtained from regional boards at Karolinska Institutet and the University of Lund.

The study included all patients with diagnosed AF, identified by the presence of the ICD-10 code (10th version of the WHO’s International Classification of Diseases) for atrial fibrillation (I48) in the Swedish Hospital register from January 1 1998 until December 31 2006.

We excluded individuals with a diagnosis of dementia during January 1 1998–December 31 2006; a new diagnosis of hypothyroidism between January 1 2007 and December 31 2015; a diagnosis of hyperthyroidism or thyroid cancer during January 1 1998–December 31 2006, and with dispensed prescription of levothyroxine on two or more occasions in these diagnoses during July 1 2005–December 31 2006; individuals below 45 years of age at January 1 2007; and individuals with a dispensed prescription of levothyroxine on only one occasion during July 1 2005–December 31 2006. In total, 156,104 individuals (89,251 men and 66,853 women), aged ≥ 45 years were included (see Supplementary Fig. 1!).

The exposition was at least two dispensed prescription of levothyroxine (H03AA01) during July 1 2005–December 31 2006, aged 45 years of age or above and alive at January 1 2007. Individuals without a dispensed prescription were used as reference group.

The outcome was defined as a diagnosis of dementia according to ICD-10 classification in electronic patient records from hospitals, or from the Death register, and was used as primary outcome (F00 Alzheimer’s dementia, F01 Vascular dementia, F02 Dementia in other diseases classified elsewhere, F03 Unspecified dementia, F10.7A Alcohol dementia, F10.97Alcohol use, unspecified with alcohol-induced persisting dementia or G30 Alzheimer’s disease). We categorized dementia as Alzheimer’s dementia (AD; F00 and G30), vascular dementia (VaD; F01) or other types of dementia (remaining diagnoses as listed above). For the primary outcome, time to diagnosis of dementia between January 1 2007 and December 31 2015 was registered. Individuals were also divided into the following pre-specified age-groups: 45–64, 65–84, and > 85 years. Individuals < 45 years of age were excluded. Educational level was categorized according to pre-specification as ≤ 9 years (partial or complete compulsory schooling), 10–12 years (partial or complete secondary schooling) and > 12 years (college and/or university studies). Country of birth was classified as born in Sweden or foreign born. Marital status was classified as married, unmarried, divorced or widowed. The neighborhood socioeconomic status (SES) areas were categorized into three groups according the neighborhood index: > one standard deviation (SD) below the mean (high SES or low deprivation level), > one SD above the mean (low SES or high deprivation level), and within one SD of the mean (middle SES or deprivation level).

The following related disorders were used as covariates (with ICD-10 codes): hypertension (I10–15); coronary heart disease (CHD; I20–25); congestive heart failure (CHF; I50 or I110); cerebrovascular diseases (CVD; I60–69); obesity (E65–E68); diabetes mellitus (E10–14); chronic obstructive pulmonary disease (COPD; J40–J47); depression (F32–F34, F38–F39); anxiety disorders (F40–41); and alcoholism and related disorders (F10 and K70).

Cardiovascular medication was recorded, with at least two dispensed prescription of the following drugs: loop diuretics (C03C), thiazides (C03A, C03B, C03E, C09B and C09DA), potassium-saving agents (C03D), beta-1-selective beta-blockers (C07AB and C07FB), non-selective beta-blockers (C07AA and C07AG), heart-active calcium channel blockers (C08DA), vessel-active calcium channel blockers (C08CA and C08DB), ACE inhibitors (C09AA and C09BA), angiotensin-receptor blockers (ARBs; C09CA, C09DA and C09DB), statins (C10AA) and anticoagulants (B01AA).

Descriptive data were shown as numbers and percentages, and with differences between individuals with incident dementia vs those without dementia shown as *p* values obtained from chi-square test. Cox regression with hazard ratios (HRs) and 95% confidence interval (95% CI), using time to dementia as the outcome, was used in follow-up analyses for patients with levothyroxine treatment compared to those without this, for all individuals and also categorized by sex. We used the following regression models: Model 1 adjusted for age; Model 2 adjusted for age, educational level, immigrant status, marital status, and neighborhood deprivation; Model 3 as Model 2 + comorbidities; and Model 4 as Model 3 + cardiovascular medication. In subgroup analyses, we analyzed dementia diagnosed as AD, VaD or other types of dementia. Furthermore, as risk factors for dementia differ over age, we also analyzed patients in the following age strata: 45–64 years of age, 65–84 years of age, and 85 years of age and above.

A two-sided *p* value of < 0.01 was considered statistically significant for variables at baseline owing to multiple testing, and < 0.05 for variables in the regression analyses. All analyses were performed in SAS 9.4.

## Results

Characteristics of the study population (*n* = 156,104 individuals; 89,251 men and 66,853 women) are shown in Table 1. In total, 9054 dementia diagnoses were recorded (5.8%), 4486 among men (5.0%) and 4568 (6.8%) among women. Mean follow-up was 5.4 years in the total sample, in the age-group 45–64 years 7.4 years, in the age-group 65–84 years 5.7 years, and in the age-group 85 years and above 3.0 years. Total person-years were in the whole sample 836,270 years and, in the age-groups 45–64, 65–84 and 85 years and above, 199,429, 525,186 and 111,655 years, respectively. Among individuals with dementia there were more women. Individuals with dementia were also older, had lower educational level, were less often married, had more often registered diagnoses of CHD, stroke, and depression, less often registered diagnoses of obesity and COPD, and were less often treated with anticoagulants. There were only small differences in regard to dispensed cardiovascular drugs, with higher rate of loop diuretics and lower rate of ARBs among dementia patients (Supplementary Table 1).

**Table 1:** Baseline data of patients with atrial fibrillation and with or without levothyroxine treatment, and number of incident cases of dementia during follow-up

	Population		Dementia events		Without dementia		*P* value*
	No.	%	No	%	No	%	
Total population	156,104		9054		147,050		
Exposition							< 0.001
Levothyroxine treatment	12,978	8.3	878	9.7	12,100	8.2	
No levothyroxine treatment	143,126	91.7	8176	90.3	134,950	91.8	
Gender							< 0.001
Males	89,251	57.2	4486	49.5	84,765	57.6	
Females	66,853	42.8	4568	50.5	62,285	42.4	
Age (years)							< 0.001
45–64	26,919	17.2	209	2.3	26,710	18.2	
65–84	91,732	58.8	6175	68.2	85,557	58.2	
≥ 85	37,453	24.0	2670	29.5	34,783	23.7	
Educational level							< 0.001
≤ 9	76,264	48.9	4719	52.1	71,545	48.7	
10–12	36,017	23.1	2023	22.3	33,994	23.1	
> 12	43,823	28.1	2312	25.5	41,511	28.2	
Immigrant status							0.2674
Born in Sweden	140,224	89.8	8102	89.5	132,122	89.8	
Foreign born	15,880	10.2	952	10.5	14,928	10.2	
Marital status							< 0.001
Married	85,474	54.8	4322	47.7	81,152	55.2	
Not married	70,630	45.2	4732	52.3	65,898	44.8	
Neighborhood deprivation							< 0.001
Low	17,347	11.1	966	10.7	16,381	11.1	
Middle	78,938	50.6	4306	47.6	74,632	50.8	
High	18,670	12.0	1085	12.0	17,585	12.0	
Unknown	41,149	26.4	2697	29.8	38,452	26.1	
Hospital diagnosis							
Hypertension	43,854	28.1	2592	28.6	41,262	28.1	0.2428
CHD	43,111	27.6	2646	29.2	40,465	27.5	< 0.001
Heart failure	40,502	25.9	2294	25.3	38,208	26.0	0.1734
Stroke	25,610	16.4	1808	20.0	23,802	16.2	< 0.001
Obesity	1454	0.9	31	0.3	1423	1.0	< 0.001
Diabetes	17,623	11.3	1012	11.2	16,611	11.3	0.7289
COPD	10,998	7.0	555	6.1	10,443	7.1	< 0.001
Depression	3505	2.2	314	3.5	3191	2.2	< 0.001
Anxiety	2172	1.4	148	1.6	2024	1.4	0.0418
Alcoholism and related disorders	2932	1.9	182	2.0	2750	1.9	0.3407

*CHD* coronary heart disease, *COPD* chronic obstructive pulmonary disease

*P value was calculated based on chi-square test

Tables 2, 3 and 4 show Cox regression models for incident dementia for subjects with levothyroxine treatment vs. subjects without treatment, categorized by sex and also for men and women combined. Table 2 shows results in four models, while in Tables 3 and 4 only fully adjusted models are shown. Table 3 is categorized by age-groups, and Table 4 is categorized by type of dementia. We did not observe any association of levothyroxine therapy with incident dementia.

**Table 2:** Cox regression of dementia diagnosis among men (*n *= 89,251) and women (*n* = 66,853) with atrial fibrillation HR and with or without levothyroxine treatment

	Model 1		Model 2		Model 3		Model 4	
	HR	95% CI	HR	95% CI	HR	95% CI	HR	95% CI
Men								
Levothyroxine treatment	1.04	0.89–1.22	1.03	0.87–1.20	1.01	0.86–1.19	1.01	0.86–1.19
No treatment	Ref		Ref		Ref		Ref	
Women								
Levothyroxine treatment	1.07	0.98–1.16	1.04	0.96–1.14	1.04	0.96–1.13	1.04	0.95–1.13
No treatment	Ref		Ref		Ref		Ref	
All^a^								
Levothyroxine treatment	1.07	0.99–1.15	1.04	0.97–1.12	1.03	0.96–1.12	1.03	0.96–1.11
No treatment	Ref		Ref		Ref		Ref	

*Model 1* adjusted for age, *Model 2* adjusted for age, educational level, immigrant status, marital status, and neighborhood deprivation, *Model 3* Model 2 + comorbidities, *Model 4* Model 3 + cardiovascular medications

^a^Gender was added in the Model 2, Model 3, and Model 4

**Table 3:** Cox regression of dementia diagnosis among men (*n* = 89,251) and women (*n* = 66,853) with atrial fibrillation HR and with or without levothyroxine treatment in different age-groups in fully adjusted model

	Aged 45–64 years		Aged 65–84 years		Aged 85 + years	
	HR	95% CI	HR	95% CI	HR	95% CI
Men						
Levothyroxine treatment	1.84	0.68–5.00	1.01	0.84–1.22	0.97	0.71–1.34
No treatment	Ref		Ref		Ref	
Women						
Levothyroxine treatment	0.71	0.26–1.95	1.06	0.95–1.18	0.99	0.86–1.14.
No treatment	Ref		Ref		Ref	
All^a^						
Levothyroxine treatment	1.05	0.51–2.15	1.05	0.95–1.15	0.99	0.87–1.13
No treatment	Ref		Ref		Ref	

*Full Model* adjusted for age, educational level, immigrant status, marital status, neighborhood deprivation, comorbidities, and cardiovascular medications

^a^Gender was added in the fully adjusted model

**Table 4:** Cox regression of dementia diagnosis among men (*n* = 89,251) and women (*n* = 66,853) with atrial fibrillation HR and with or without levothyroxine treatment in different dementia groups in fully adjusted model

	Alzheimer’s disease		Vascular dementia		Others	
	HR	95% CI	HR	95% CI	HR	95% CI
Men						
Levothyroxine treatment	1.05	0.78–1.43	1.21	0.92–1.61	0.87	0.68–1.12
No treatment	Ref		Ref		Ref	
Women						
Levothyroxine treatment	1.04	0.88–1.23	1.03	0.86–1.24	1.05	0.93–1.18
No treatment	Ref		Ref		Ref	
All^a^						
Levothyroxine treatment	1.05	0.91–1.21	1.08	0.93–1.26	1.01	0.91–1.12
No treatment	Ref		Ref		Ref	

*Full Model* adjusted for age, educational level, immigrant status, marital status, neighborhood deprivation, comorbidities, and cardiovascular medications

^a^Gender was added in the fully adjusted model

## Discussion

The main finding of this study was that levothyroxine treatment in AF patients was not associated with different risk of incident dementia. This was true for men and women, and also when divided by age-groups and type of dementia.

Our results seem to confirm the conclusion in an earlier review that there is no association between cognitive disturbances and dementia due to thyroid dysfunction [18]. However, there are studies challenging this conclusion. In a study of AF patients in Swedish primary care, a lower risk of dementia was found in women with hypothyroidism and levothyroxine treatment, while no such association was found in men and women with levothyroxine treatment but without a diagnosis of hypothyroidism [19]. Besides, in animal models an association between treatment with thyroid hormones and an improved brain function has been shown [6, 7]. In contrast, there are other studies showing an increased risk of cerebrovascular diseases associated with treated hypothyroidism [1, 2]. The reason for this discrepancy between studies is unclear, even if differences in methodology used, and populations included, could contribute to this. However, more studies on this topic are needed, including studies with levels of thyroid hormones.

In regard to comorbidities, individuals with incident dementia have been shown to more often be registered with some diagnoses, also known as risk factors for dementia, i.e. CHD [20], stroke [21], diabetes [22, 23], and depression [24, 25]. Some cardiovascular drugs, including anticoagulant treatment and some antihypertensive drugs, have also been shown to be associated with a lower risk of dementia among AF patients [26, 27]. However, adjusting for these factors only changed the HR estimates marginally.

There are several limitations of this study which must be kept in mind when interpreting the results. We did not have access to levels of thyroid hormones in the study, which could be of importance in further studies. Thus, we could not identify patients with over- or under-treatment with levothyroxine. As this is an observational study, there may be residual confounding present, and the result may be affected by biases such as competing risks, i.e. patients may die due to other causes before getting a dementia diagnosis, or survival treatment selection bias [28]. Besides, as dementia develops over a period of many years the results of the setting of the diagnosis in relation to time may differ. We included patients with AF registered in hospital care, however, both from in-patient clinics and open care. Another study showed that 12% of all registered AF patients in Stockholm County were only registered with a diagnosis in primary health care, and 9% in ambulatory specialist care, including hospital out-patient clinics [29]. Clinical diagnoses were taken from hospital care, while most diagnosis, i.e. hypertension, diabetes, COPD, depression and anxiety, will be underrepresented as most patients are cared for these diseases in primary care [30]. We included patients with levothyroxine treatment recorded on at least two occasions, to ensure that this was a long-term treatment. However, we had data neither on levothyroxine treatment before 2005 nor on treatment duration. Our intention was to study patients with levothyroxine treatment with and without a diagnosis of hypothyroidism, but as most patients with this diagnosis are cared for in primary care only, a smaller part was identified with this diagnosis (1711 individuals or 13.8% of all with levothyroxine treatment). In the present study, we only had access to diagnoses of hypothyroidism in the Inpatient Register, while no diagnoses from primary care are included, and we had no possibility to identify patients with levothyroxine treatment without a diagnosis of hypothyroidism. In an earlier, smaller study (*n* = 12,057) of AF patients in primary care, as much as 35% of the patients, could not be identified with a diagnosis of hypothyroidism [19]. Moreover, AF could not be classified as paroxysmal, persistent or permanent and heart rhythm could not be classified as sinus rhythm or fibrillation rhythm. Additionally, we had not access to data on kidney function. Besides, we have chosen not to report results of co-morbidities and cardiovascular medications used for adjustment, as we are analyzing these factors in other sub-studies, and including them here would go far beyond the main topic of the actual study.

In conclusion, we found no significant differences in incident dementia among AF patients with or without levothyroxine treatment. However, as previous studies show divergent results, it is of interest to perform further studies within this area. Furthermore, it would also be of interest to study the general potential effect of levothyroxine in relation to incident dementia, and not restrict future studies to patients with AF only. Yet, levothyroxine seems to be safe regarding dementia in patients with AF and also in combination with many cardiovascular pharmacotherapies.

## Electronic supplementary material

Below is the link to the electronic supplementary material.

Supplementary material 1 (DOCX 62 kb)
