# Supplementary material for: Levothyroxine treatment and incident dementia in adults with atrial fibrillation
Source: Aging Clin Exp Res. 2019 May 22;32(3):433–9. doi: 10.1007/s40520-019-01217-3 (PMC6872912; doi:10.1007/s40520-019-01217-3)
Supplement: Supplementary file 1 — Supplementary material 1 (DOCX 62 kb) [file 40520_2019_1217_MOESM1_ESM.docx]

**Supplementary Figure 1.** Flowchart over eligible, excluded and included patients in the study of AF patients with levothyroxine treatment and incident dementia.

| **Supplementary Table 1. Baseline data of patients with atrial fibrillation and with or without levothyroxine treatment, and number of incident cases of dementia during follow-up** | | | | | | | | | | |
| --- | --- | --- | --- | --- | --- | --- | --- | --- | --- | --- |
|  | Population | |  | Dementia events | |  | Without dementia | |  |  |
|  | No. | % |  | No | % |  | No | % |  | P-value* |
| **Total population** | 156104 |  |  | 9054 |  |  | 147050 |  |  |  |
| **Cardiovascular medication** | 152349 | 97.6 |  | 8879 | 98.1 |  | 143470 | 97.6 |  | 0.0025 |
| a.   Loop diuretics (C03C) | 11931 | 7.6 |  | 798 | 8.8 |  | 11133 | 7.6 |  | <0.001 |
| b.  Thiazides (C03A, C03B, C03E, C09B C09DA) | 6434 | 4.1 |  | 402 | 4.4 |  | 6032 | 4.1 |  | 0.1163 |
| c.  Potassium-saving agents (C03D) | 3474 | 2.2 |  | 215 | 2.4 |  | 3259 | 2.2 |  | 0.3214 |
| d.  Beta-1-selective beta-blockers (C07AB and C07FB) | 43777 | 28.0 |  | 2528 | 27.9 |  | 41249 | 28.1 |  | 0.7898 |
| e.  Non-selective beta-blockers (C07AA and C07AG) | 8585 | 5.5 |  | 447 | 4.9 |  | 8138 | 5.5 |  | 0.0156 |
| f.  Heart-active calcium channel blockers (C08DA) | 1952 | 1.3 |  | 103 | 1.1 |  | 1849 | 1.3 |  | 0.3195 |
| g.  Vessel-active calcium channel blockers (C08CA and C08DB) | 12411 | 8.0 |  | 782 | 8.6 |  | 11629 | 7.9 |  | 0.0128 |
| h.  ACE inhibitors (C09AA and C09BA) | 27874 | 17.9 |  | 1558 | 17.2 |  | 26316 | 17.9 |  | 0.0971 |
| i.  ARBs (C09CA, C09DA and C09DB) | 10006 | 6.4 |  | 500 | 5.5 |  | 9506 | 6.5 |  | <0.001 |
| j.  Statins (C10AA) | 19097 | 12.2 |  | 1110 | 12.3 |  | 17987 | 12.2 |  | 0.9374 |
| k. Anticoagulants (B01AA) | 9607 | 6.2 |  | 582 | 6.4 |  | 9025 | 6.1 |  | 0.2639 |
| ARBs: Angiotensin receptor blocking agents. | | | | | | | | | | |
| *: P-value was calculated based on Chi-square test. | | | | | | | | | | |
